# *HPRT* Mutations in Lymphocytes from 1,3-Butadiene-Exposed Workers in China

**DOI:** 10.1289/ehp.10353

**Published:** 2007-11-12

**Authors:** Shengxue Liu, Lin Ao, Bing Du, Yanhong Zhou, Jian Yuan, Yang Bai, Ziyuan Zhou, Jia Cao

**Affiliations:** 1 Department of Hygienic Toxicology, Preventive Medical College, Third Military Medical University, Chongqing, People’s Republic of China; 2 Institute of Occupational Disease, Yangzi Petrochemical Products Company, Nanjing, People’s Republic of China

**Keywords:** 1,3-butadiene, BD, exon deletion, *HPRT* gene, lymphocyte, occupational exposure

## Abstract

**Background:**

1,3-Butadiene (BD) is an important industrial chemical and an environmental and occupational pollutant. The carcinogenicity of BD in rodents has been proved, but its carcinogenic and mutagenic molecular mechanism(s) are not fully elucidated in humans.

**Objectives:**

In the present study, we compared the mutation frequencies and exon deletions of BD-exposed workers with that of control subjects in China to identify the characteristic mutations associated with BD exposure in the human *HPRT* (hypoxanthine–guanine–phosphoribosyltransferase) gene.

**Methods:**

Seventy-four workers exposed to BD via inhalation and 157 matched controls were evaluated in Nanjing, China. Molecular analysis of *HPRT* mutant T lymphocytes from BD-exposed workers and nonexposed control subjects was conducted to identify changes in the structure of the *HPRT* gene. A total of 783 *HPRT* mutants were analyzed by multiplex polymerase chain reaction, in which 368 *HPRT* mutants were isolated from BD-exposed workers and 415 mutants from control subjects.

**Results:**

The BD-exposed workers showed a higher mutation frequency (18.2 ± 9.4 × 10^−6^) than the control subjects (12.7 ± 7.3 × 10^−6^), but the difference was not significant (*p* > 0.05). The frequency of exon deletions in BD-exposed workers (27.4%) was significantly higher than that in control subjects (12.5%) (*p* < 0.05), which mainly included multiplex exon deletions (2–8 exons).

**Conclusions:**

The results of the present study suggest that BD should increase the frequency of large deletions of *HPRT* gene in human lymphocytes This change confirms and supports the previous findings in BD-exposed workers.

1,3-Butadiene (BD) is a highly volatile four-carbon chemical [C_4_H_6_; Chemical Abstracts Service (CAS) no. 106–99–0] made from petroleum processing. It is a colorless, flammable gas that is widely used in the production of rubber and thermoplastic resins (Morrow 1990). Its annual worldwide production is approximately 12 billion pounds, with 1.6 billion pounds produced in China (Cui 2003). Following the United States, China is now the second leading producer and consumer of BD. While useful for industry, BD is also an environmental air pollutant commonly found in automobile exhaust and cigarette smoke.

BD is a potent carcinogen at several sites in mice and rats after inhalation exposure. Results from exposures in rodent studies indicate species differences in carcinogenic susceptibility between mice and rats: B6C3F_1_ mice were observed to be more sensitive to BD-induced carcinogenicity than Sprague-Dawley rats (Huff et al. 1985; Glaister and Owen 1990; Owen et al. 1987). While the species and individual susceptibilities to DNA damage appear to differ greatly among mice, rats, and humans, it has been strongly suggested that the mutagenicity and carcinogenicity of BD are related to its metabolic activation in several DNA-reactive intermediates, including 1,2-epoxy-3-butene (EB), 1,2,3,4-diepoxybutane (DEB), and 3,4-epoxy-1,2-butanediol (EB-diol) (Richardson et al. 1999).

Although research has shown that BD is a potent animal carcinogen, only a few studies have indicated that BD is a probable human carcinogen. In several epidemiologic studies, occupational exposure to BD is believed to be associated with excess mortality from lymphatic and hematopoietic cancers (Delzell et al. 1996; Divine and Hartman 1996). Overall, the epidemiologic findings in BD-exposed workers suggest, but do not prove, that it is a human carcinogen (Zhang et al. 2004). BD had been classified in Group 2A (probably carcinogenic to humans) by the International Agency for Research on Cancer (IARC 1992), but several regulatory agencies have recently considered raising its status to Group 1 (carcinogenic to humans) on the basis of the emerging data (Acquavella and Leonard 2001; IARC 1999; Morrow 2001). In a recent study of mortality among workers in the North American synthetic rubber industry, Cheng et al. (2007) found the presence of a causal relationship between high cumulative exposure and high-intensity exposure to BD and leukemia. The weight of this evidence led to the recent classification of BD as a Group 1 known human carcinogen by the IARC Working Group (IARC, in press).

Measuring mutation frequency (MF) at the hypoxanthine–guanine–phosphoribosyltransferase (HPRT) locus as an intermediate biomarker of BD carcinogenicity could be a powerful complement to traditional methods based on mortality and cancer incidence. MF of the *HPRT* gene as a biomarker of genotoxicity has been investigated in BD-exposed humans, but the findings are inconsistent. Three studies of BD-exposed workers in a Texas facility conducted by one laboratory indicated *HPRT* mutations in blood lymphocytes using the autoradiographic assay (Ammenheuser et al. 2001; Ward et al. 1994, 1996, 2001). In contrast, studies by Hayes et al. (1996) and Tates et al. (1996) using the T-cell cloning assay, failed to find significant increases in MF in blood lymphocytes of BD-exposed Chinese and Czech workers, even though BD exposure concentrations were similar to those detected in the Texas studies. Furthermore, no increase in MF was found in the studies by Albertini et al. (2001) using both the autoradiographic and T-cell cloning assays followed by the T-cell cloning assay in the follow-up *HPRT* mutation study (Albertini et al. 2007). It is important to note that in the early studies, the *HPRT* autoradiographic assay was consistently used, whereas in later and follow-up studies, the T-cell cloning assay or a combination of the two assays was used. It is possible that differences in the sensitivity of the methods and the target cells may have resulted in contradictory findings. For cytogenetic effects, the Czech study found a significant increase in chromosomal aberration frequencies and sister chromatid exchanges (SCEs) in the BD-exposed workers (Sram et al. 1998). However, subsequent studies by Sram et al. (2004) and Albertini et al. (2001, 2003, 2007) failed to confirm this finding. The current consensus on existing data is that BD does not cause cytogenetic effects in humans, most likely because DEB is not formed at sufficient levels in humans to play a role in its carcinogenicity. Thus, the remaining open question is whether BD is mutagenic in humans.

At the BD exposure levels encountered in modern industry, a recent large study failed to demonstrate either induction of *HPRT* mutations or cytogenetic changes, even though measurable levels of electrophilic BD metabolites were produced *in vivo* (Albertini et al. 2007). Studies of workers at higher exposure levels are required to clarify the question of genotoxicity, which is the important qualitative non-tumor end point for making human cancer risk assessments for BD (Albertini et al. 2003). In the present study, we analyzed a large population of BD-exposed workers and nonexposed control subjects from Yangzi Petrochemical Products Company in Nanjing, China, to determine the MF of the *HPRT* gene in lymphocytes. We then compared the differences in the exon deletions to help us understand the mechanisms responsible for the mutagenicity of BD.

## Materials and Methods

### Subject selection and specimen collection

The large population study was initiated in 2002 at Yangzi Petrochemical Products Co. in Nanjing, China. All subjects worked at the alkenes plant of this company. A total of 237 subjects were included in the study. Among them, 80 subjects from the BD production workshop were categorized into the BD-exposed group, of which 74 subjects (92.5%) finished the evaluations. For comparision, 157 subjects from the administrative office and the vapor workshop of the same plant were enrolled as members of the control group, and all the control subjects (100%) finished the evaluations. The subjects in the BD-exposed group and the control group were matched for age and sex.

Questionnaires and blood samples for all subjects were accompanied by regular physical examinations once every 2 years at the Yangzi Employee Hospital. An informed consent document was signed by each subject after the procedures and the purpose of the study were explained. All subjects were asked to complete a questionnaire to gather information on sex; age; employment history; chemical exposure; history of the use of tobacco, alcohol, and caffeine-containing beverages; recent illnesses; general health; and medications, etc.

A 15-mL blood sample was collected from each subject in the Yangzi Employee Hospital. Blood samples were stored at room temperature in an insulated container and were delivered to the laboratory within 12 hr of collection.

### Exposure assessment

Because of the production process for BD and the activities of workers during the work shift, 10 locations in the BD production workshop were chosen for air sampling, including the areas for procedure control, the analytical laboratory, product storage, and the pipeline. The individuals in the BD-exposed group worked 8 hr/day, 4 days/week. Except for regular sampling (once every 4 hr) and circuit inspection (once per hour), the workers in the BD production workshop stayed in the procedure control center or the analytical laboratory while they were on duty. According to the typical workers’ routines, it was estimated that the duration of time spent in the product storage and pipeline areas was no more than 1 hr/day, or 300 hr/year, for each worker. For comparison with the production locations, two locations—one in the administrative office and one in the vapor workshop of the same plant—were chosen for air sampling in the control group.

For each sampling location, an air sampler (Gillian Instrument Corp., Wayne, NJ, USA) collected air samples once a day for 3 consecutive days. The air was drawn through charcoal tubes using a sampling pump, and a total of 4 L of air were collected at a flow rate of 0.2 L/min for each sample. After sampling, each charcoal tube collected was desorbed in dichloromethane alkyl, and the samples were analyzed quantitatively for BD using a gas chromatograph fitted with a flame inozation detector (Beijing Analytical Instrument Factory, Beijing, China) according to the standard operation procedure.

### *HPRT *mutation assay and mutant expansion

We selected the T-cell cloning assay as the *HPRT* mutation assay for our study; the method has been described previously (Ma et al. 2000). Briefly, Ficoll reagent (Haoyang Biological Manufacture Co., Tianjin, China) was used to isolate the peripheral lymphocytes from whole blood by density centrifugation. The isolated lymphocytes were stimulated for 48 hr in RPMI 1640 medium supplemented with 2 μg/mL purified phytohemagglutinin (PHA-M; Gibco/BRL Life Technologies Inc., Rockville, MD, USA), 20% HL-1 medium (Bio-Whittaker Inc., Walkersville, MD, USA), and 10% bovine calf serum (BCS; Gibco/BRL Life Technologies Inc.). The lymphocytes were then counted and seeded at 1 × 10^4^ viable cells per well in round-bottomed 96-well micro-well plates with RPMI 1640 medium supplemented with reduced PHA-M (1 μg/mL), 10% human T-stim (Collaborative Biomedical Products, Bedford, MA, USA), 20% HL-1 medium, and 15% BCS. The cells were cultured together with an equal number of irradiated lymphoblastoid feeder cells (irradiation was 50 Gy Cobalt-60). To determine the cloning efficiency (CE), lymphocytes were plated at densities of one and two cells per well in the absence of 6-thioguanine (6-TG). To determine the MF, the remaining cells were plated at 1 × 10^4^ viable cells per well in the presence of 6-TG (Sigma-Aldrich Chemical Co., St. Louis, MO, USA). All the plates were incubated at 37°C in a humidified 5% CO_2_ atmosphere for 10–14 days to determine CE, or to select the *HPRT* mutant clones and determine MF. CE was calculated using Poisson distribution analysis: *CE* = −ln *P*_0_/*N*, where *P*_0_ was the fraction of wells negative for colony growth and *N* was the average number of cells originally inoculated per well by limiting dilution. The thioguanine-selected CE divided by the mean unselected CE yields the MF.

Mutant lymphocyte clones were identified with an inverted microscope (Olympus, Tokyo, Japan). All the mutant clones were selected for expansion, and the culture medium for expansion was the same as that for the initial culture in 96-well plates. Mutant clones were expanded first from one well of 96-well plates to 24-well plates, then to 6- or 12-well culture plates. Only the clones that expanded successfully to ≥ 1 × 10^6^ cells were collected for DNA isolation, and the aliquots were frozen for further molecular analysis.

### Isolation of DNA

All wells of an expanded mutant clone were pooled and harvested for the isolation of DNA. A wizard genomic DNA purification kit (Promega Corp., Madison, WI, USA) was used to isolate DNA from cells; the isolation was performed according to the procedure of Ma et al. (2000). Simultaneously, normal lymphocytes were collected to isolate DNA to serve as controls for the further molecular analysis.

### Multiplex PCR amplification of the* HPRT *gene

Exon loss and changes in the size of each exon were identified by multiplex polymerase chain reaction (PCR) directly, and these changes were defined as exon deletions of the *HPRT* gene. If the number of exon deletions were ≥ 2 (2–9 exons), they were considered large deletions. All expanded *HPRT* mutant clones were analyzed by a multiplex PCR assay to detect exon deletions.

Multiplex PCR amplification of the *HPRT* gene was performed as previously described (Liu et al. 2003), with slight modifications. Nine *HPRT* exons were amplified simultaneously using eight pairs of PCR primers; exons 7 and 8 were amplified as a single PCR product. For amplification of *HPRT* exons, the genomic DNA template (36–50 ng) was mixed with 50 pmol of each primer pair in a total reaction volume of 50 μL, containing 50 mM KCl, 10 mM Tris–HCl (pH 8.8), 0.3–1.05 mM MgCl_2_, 0.2 mM deoxynucleotide triphosphate, and 2.5 U of Amplitaq DNA polymerase (Shenggong Chemical Co., Shanghai, China). After initial denaturation of the template DNA at 98°C for 7 min, a total of 40 PCR cycles were performed with denaturation at 94°C for 1.5 min, annealing at 52°C for 1.5 min and extension at 72°C for 2.0 min. Exon 1 was synthesized individually with modified conditions: a total of 30 PCR cycles were performed with denaturation at 95°C for 0.5 min, annealing at 64°C for 1.0 min and extension at 72°C for 1 min. The last cycle was finished with a 7-min extension at 72°C. The PCR product (10 μL) was analyzed by 3% agarose gel electrophoresis or 5% polyacrylamide gel electrophoresis.

### Statistical analysis

The differences in the *HPRT* mutant frequency and cloning efficiency between the two study groups were analyzed by the Student *t*-test. These statistical evaluations were performed with SPSS 11.0 for Windows (SPSS Inc., Chicago, IL, USA).

## Results

### Exposure assessment

**Figure 1:**
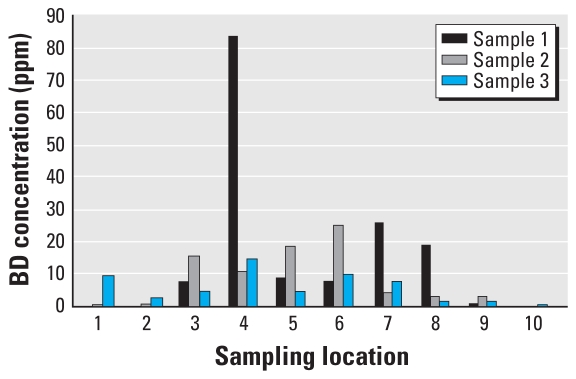
BD exposure dose at each sampling location of the BD-exposed group.

In the present study the minimum exposure to BD that could be measured with our equipment was 0.2 ppm. All 30 samples evaluated at the 10 locations of the BD-exposed group ranged from 0 to 83.1 ppm; among them, four samples could not be measured, which meant their values were < 0.2 ppm (Figure 1; Table 1). The sample with the highest concentration of BD (83.1 ppm) was collected from the sewer exit in the workshop and was only observed once. When all data from three samplings were considered, the average BD exposure was 9.7 ± 15.7 ppm. If the highest value was ignored, the other 29 samples ranged from 0 to 25.8 ppm, and the average value was 7.2 ± 7.6 ppm in the exposed areas. All samples evaluated at the control group locations were below the minimum exposure that could be measured (< 0.2 ppm). The data of BD exposure in this study were consistent with the historical records from 1991 to 2001 in this workshop.

### Study subjects and MF

On the basis of the information collected using the questionnaire, BD-exposed workers (*n* = 74) and control subjects (*n* = 157) had similar demographic characteristics (Table 2), including sex, age, and duration of employment. The mean age for BD-exposed workers and control subjects was 30.7 and 34.8 years of age, respectively. None of the female subjects in either group smoked, but the proportions of male smokers were 36.5% in the BD-exposed group and 33.8% in the control group, respectively. There were no significant differences found in smoking, consumption of alcohol, or consumption of caffeine-containing beverages between BD-exposed workers and control subjects.

The lymphocyte cloning efficiencies and the frequencies of mutants of the study population are listed in Table 3. There was a non-significant difference in CE between the BD-exposed workers and control subjects. The MF of BD-exposed workers was 18.2 ± 9.4 (×10^−6^), which was higher than the 12.7 ± 7.3 (×10^−6^) for the control subjects, but this difference was not significant (*p* > 0.05). The mean numbers of mutants per subject were 8 in the BD-exposed group and 6 in the control group.

### Exon deletions detected by multiplex PCR

**Figure 2:**
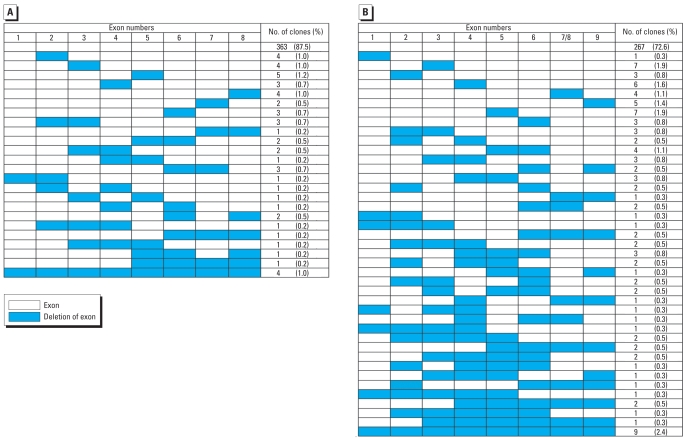
Schematic diagram of the distribution of deleted exons in mutants from BD-exposed subjects and control subjects. (*A*) Control subjects. (*B*) BD-exposed subjects.

We analyzed a total of 783 *HPRT* mutants by multiplex PCR, including 368 mutants from BD-exposed workers and 415 mutants from control subjects. Results of the exon deletions detected by multiplex PCR are summarized in Table 4. A schematic of the distribution of deleted exons in mutants is presented in Figure 2, which shows that 630 mutant clones amplified all nine exons after multiplex PCR analysis, indicating these clones with wild-type multiplex PCR patterns had potential point mutations. The frequency of exon deletions in BD-exposed workers was 27.4% (101/368), which was significantly higher than the 12.5% (52/415) in control subjects (*p* < 0.05). The types of deletion mutants can be divided into single exon deletions, multiple exon deletions (2–8 exon deletions), and mutants in which all nine exons had deletions. The increase in exon deletions in the BD-exposed group appears to be mainly the result of an increase in the proportion of multiple exon deletions (56/368, 15.2%) compared with the control group (23/415, 5.5%; *p* < 0.05). The multiple exon deletions can be further divided into continuous and discontinuous deletions, both of which had significant differences between the BD-exposed group and the control group (*p* < 0.05). In addition, results of PCR analysis showed that some of the mutants from the same subject had different mutational spectra (data not shown).

## Discussion

In 1996 Hayes et al. (1996) first reported the *HPRT* gene MF among workers exposed to BD in China. That study was conducted at Yanshan Petrochemical Products Co., one of the biggest petrochemical products corporations in China located in Beijing in the northern part of China. The present study was performed at the Yangzi Petrochemical Products Co., another large petrochemical products corporation located in Nanjing in the eastern part of China. It should be noted that the solvent for producing BD is different between these two plants—acrylonitrile (ACN) in Yanshan Petrochemical Products Co. and dimethylformamide (DMF) in Yangzi Petrochemical Products Co. In addition, the BD production equipment in the present study was imported from Japan and has been used to produce BD since 1987.

According to records kept by the facility that documented BD air concentrations, the exposure levels detected in 1991 in this workshop ranged from 0 to 4.4 ppm. In 1996 there were 9 samples that ranged from 3.1 to 24.9 ppm among the 27 total samples collected from 9 locations of the same workshop. The other 18 samples had BD levels below the limit of detection (LOD; the minimum exposure dose that could be measured depended on the analytical method. In China the LOD was about 0.4 ppm when sampling with syringe, but 0.2 ppm when sampling with charcoal tube). In 1998 another study of BD exposure was carried out in the same workshop. In this study there were a total of 60 samples collected from 20 locations with exposure levels ranging from 0 to 6.0 ppm. In the present study, when data from all three samplings were considered, the average value of BD detected was 9.7 ppm. If we excluded the highest value from the sewer exit location in the workshop, the other 29 samples ranged from 0 to 25.8 ppm, and the average value of BD was 7.2 ppm (Table 1). These BD exposure data are consistent with the historical records for the same workshop recorded from 1991 to 2001 described above. Comparing this BD exposure data with other studies, our data indicate that the BD level is higher than that detected in the Yanshan Petrochemical Products Co. (Hayes et al. 1996), in which air levels of BD ranged from 1–45 ppm, and the median level was 2.0 ppm during routine activities. However, our data showed much higher BD levels than those detected in other BD-producing plants, one in Texas, the other near Prague in the Czech Republic. In the Texas plant the BD exposure levels found for workers in the first study were 3.5 ± 7.5 ppm in the high-exposure areas (Ward et al. 1994). With the improvements made in the plant to reduce the BD exposure, the average exposure decreased to 1.65 ± 0.52 ppm in the high-exposure areas and to 0.07 ± 0.03 ppm in the low-exposure areas, with a median exposure level of 0.41 ppm (Ward et al. 2001). In the plant near Prague the mean exposure level was 1.76 ppm, and the individual exposure levels ranged from 0.012 to 19.77 ppm (Tates et al. 1996). Overall, exposure levels varied widely among plants of different countries. We presume that BD exposure levels depend primarily on the quality of the equipment used to produce BD, the level of automation present in the plant, the equipment running time, and length of time the operators worked in the plant. Our data showed that the alkenes plant of the Yangzi Petrochemical Products Co. in China has one of the highest BD exposure levels among several reported BD production plants.

Although BD had been identified as a rodent carcinogen, the evidence supporting a correlation between BD exposure and *HPRT* MF in humans was inconsistent. In this study we found an increase in *HPRT* gene MFs between the BD-exposed workers (18.2 ± 9.4 × 10^−6^) and the control subjects (12.7 ± 7.3 × 10^−6^) by using the T-cell cloning assay. This 43.17% difference was, however, not statistically significant. Our results were consistent with those of previous studies in BD-exposed Chinese workers under lower exposure levels of BD using the same cloning assay (Hayes et al. 1996; Zhang et al. 2004). After adjustment by multiple regression analysis for age, sex, and cloning efficiency, Hayes et al. (1996) found that the adjusted mean MF was 13.6 × 10^−6^ in nonexposed workers and 18.0 × 10^−6^ in BD-exposed workers, which was also not significantly different. Other groups using the T-cell cloning assay to study BD-exposed workers in the Czech Republic also failed to find an increase in *HPRT* MF, even though the level of BD exposure was similar to that used in the studies in the Texas plant (Albertini et al. 2001; Tates et al. 1996). All these studies are in contrast to the other studies using autoradiographic assays, which found a correlation between BD exposure and *HPRT* gene mutation (Ammenheuser et al. 2001; Ward et al. 1994, 1996, 2001). Ward et al. (1994) reported the MF increased significantly in these BD-exposed workers compared with control subjects who worked in nonindustrial settings. In a subsequent study (Ward et al. 2001), it was demonstrated that the MF in lymphocytes was significantly higher in the high-exposure group (10.67 ± 1.5 × 10^−6^) compared with the low-exposure group (3.54 ± 0.6 × 10^−6^; *p* < 0.001), even though improvements were made in the plant to reduce BD exposure. These results indicated that the positive findings had been observed repetitively in the series studies in Texas. It also suggested the differences between mutation assays may be one of the reasons that resulted in the difference in the effects of BD exposure.

Thus, because there were contradictory results from different plants in different countries, it was unclear whether BD induced an increase in MF. In 2001 Ward et al. (2001) reported they had conducted autoradiograghic assays on cryopreserved lymphocytes from subjects in the Czech Republic concurrently with their study of workers in Texas and found a very high MF of the *HPRT* gene in the Czech administrative workers who had volunteered to serve as nonsmoking controls for the study in Prague. It was proposed that the high MF in the nonsmoking controls was due, in part, to very high MFs in a few subjects, some of whom appeared to have had health problems or exposure to mutagens other than BD. This indicates that the selection of control subjects is an important factor that may determine whether there is any significant difference in MF of the *HPRT* gene between exposed and nonexposed subjects.

It is therefore imperative that more detailed information be considered when exposure data are collected and how it is associated with the individual subjects, including how much of the variation in MF is attributable to each variable (e.g., smoking status, health history, exposure history). In the present study, we evaluated data from BD-exposed and control subjects matched for age, sex, work duration, consumption of alcohol and caffeine-containing beverages, and smoking and found no significant differences between the groups. However, it is possible that if we had been able to stratify the BD-exposed and nonexposed populations according to the extent of exposure (some combination of measured exposure and years of work, plus years of smoking, for example), we could have observed if there was any effect that may have been lost in the pooled data. Other authors have paid more attention to these questions. In one study Ward et al. (2001) divided the BD-exposed subjects into subgroups according to smoking status and found that the MF of the *HPRT* gene was significantly higher in the high-exposure nonsmokers (8.64 ± 1.6 × 10^−6^) than in the low-exposure nonsmokers (3.46 ± 0.65 × 10^−6^). However, the highest MF of *HPRT* (13.10 ± 2.57 × 10^−6^) was observed in the high-exposure smokers. Their findings were consistent with those of Podlutsky et al. (1999), who found a statistically significant increase in the MF of the *HPRT* gene between the smoking group (26.6 ± 18.5 × 10^−6^) and the nonsmoking group (18.7 ± 12.0 × 10^−6^). These studies indicate that the MF observed in BD-exposed and nonexposed subjects is also dependent upon other factors such as diet, lifestyle, general health status, and exposure to other genotoxic substances in the ambient environment. These factors should be explored in future studies.

Despite the evidence indicating that BD has mutagenic effects, the molecular mechanism of its mutagenesis is not understood. The mutagenicity of major BD metabolites has been well documented in many *in vitro* studies, and analysis of the mutational spectra in these studies indicates that there is a distinct difference between EB- and DEB-induced mutations in the *HPRT* gene. The genotoxic potencies of BD metabolites are of the order: DEB >> EB >> EB-diol. However, there is controversy about which metabolites are important for human carcinogenicity.

Although DEB is the most potent mutagen, reports have suggested that it is not a significant factor in *in vivo* mutagenesis in humans (Bond et al. 1995). This is further corroborated by the presence of smaller quantities of DEB in rats and humans than those found in mice (Filser et al. 2001; Koc et al. 1999; Walker and Meng 2000). Finally, DEB and EB differ in their metabolism, mechanism of DNA interaction, and mutagenic potential.

In our study, we observed a statistically significant increase in the total number of *HPRT* clones with deletions in the BD-exposed workers compared with the control subjects (*p* < 0.05). The increase appeared to be primarily the result of an increase in multiple exon deletions (*p* < 0.05), including both continuous deletions and discontinuous deletions (Table 4). Our results are in agreement with the data from Ma et al. (2000), Burkhart-Schultz and Jones (1997), and Burkhart-Schultz et al. (1996). Analysis of the *in vitro* mutational spectra of DEB- and EB-induced mutants in a study by Steen et al. (1997a, 1997b) clearly revealed that a decrease in the proportion of single-base substitutions was associated with an increase in the proportion of large deletion mutations Our data and similar findings by Meng et al. (2004) indicate that it is not necessary to invoke the formation of DEB as the sole source of large exon deletions; rather, *HPRT* spectra data from rats indicate that EB-diol is a likely source of deletion mutations.

In summary the results of the exposure assessment study indicate the BD occupational exposure of workers in China is relatively high, when the data are compared with those from studies conducted of other countries. Thus, we suppose that the increases of exon deletions in the mutants from exposed workers likely represent the mutations induced by BD. In a previous study Ma et al. (2000) further used *HPRT* coding sequence analysis and compared the molecular mutational spectrum of BD-exposed workers with those of control subjects. The authors identified a lower frequency of single-base substitutions, a higher proportion of A:T → T:A transversions and ± 1 frameshift mutations in BD-exposed workers. These changes were also confirmed by Meng et al. (2004) in mice and rats. In the future, we plan to analyze more population samples and molecular mutational spectrums in order to elucidate the molecular mutational spectrum associated with butadiene exposure.

## Figures and Tables

**Table 1:** BD exposure doses in work locations of the BD-exposed and control groups.

Groups	Locations	No. of samples	No. below the minimum exposure dose that can be measured^a^	Exposure range (ppm)	χ̂± SD (ppm)	Historical record (ppm)
BD-exposed	10	30	4	0–83.1	9.7 ± 15.7	0–24.9
BD-exposed without the highest sample	10	29	4	0–25.8	7.2 ± 7.6	
Control	2	6	6	Could not be measured^a^	Could not be measured^a^	Could not be measured^a^

a The minimum exposure dose that could be measured depended on the analytical method. In this study it was 0.2 ppm.

**Table 2:** Selected demographic characteristics of study subjects.

Characteristic	Control [*n* (%)]	BD-exposed [*n* (%)]
No. enrolled	157	80
No. evaluated	157 (100)	74 (92.5)
Sex		
Male	110 (70.1)	54 (73.0)
Female	47 (29.9)	20 (27.0)
Age (years)		
20–30	62 (39.5)	32 (43.2)
30–40	87 (55.4)	39 (52.7)
≥ 41	8 (5.1)	3 (4.1)
χ̂± SD	34.8 ± 8.2	30.7 ± 6.7
Duration of employment		
≤ 5 years	61 (38.9)	31 (41.9)
> 5 years	96 (61.1)	43 (58.1)
Tobacco use	53 (33.8)	27 (36.5)
Alcohol use	41 (26.1)	19 (25.7)
Tea use	28 (17.8)	15 (20.3)

**Table 3:** Molecular analysis of the *HPRT* mutant lymphocytes.

	Control		BD-exposed	
Factor	*n* (range)	χ̂± SD	*n* (range)	χ̂± SD
Cloning efficiency (%)	157	32.0 ± 11.6	74	31.6 ± 9.2
Mutation frequency (×10^−6^)	157	12.7 ± 7.3	74	18.2 ± 9.4
Mean number of mutants per subject	6 (1–15)		8 (3–19)	

**Table 4:** Exon deletions in the *HPRT* gene of human T-lymphocytes.

Type of deletion	Control [*n* (%)]	BD-exposed [*n* (%)]
Total clones with deletions	52 (12.5)	101 (27.4)*
All exons deletion (9)	4 (1.0)	9 (2.4)
Multiple exon deletions (2–8)	23 (5.5)	56 (15.2)*
Continuous deletions	17 (4.1)	37 (10.1)*
Discontinuous deletions	6 (1.4)	19 (5.2)*
Single exon deletions (1)	25 (6.0)	36 (9.8)
Other mutations	363 (87.5)	267 (72.6)
Total clones analyzed	415 (100)	368 (100)

* Significantly increased compared with control subjects (*p* < 0.05).
